# Multi-replicon Architecture Drives the Global Accumulation of Resistance to Antimicrobials, Biocides, and Metals in IncF and IncH Plasmids

**DOI:** 10.1007/s00284-026-04819-z

**Published:** 2026-03-16

**Authors:** Hannay Crystynah Almeida de Souza, Arlen Carvalho de Oliveira Almeida, Carlos Adam ConteJunior, Pedro Panzenhagen

**Affiliations:** 1 Center for Food Analysis (NAL), Technological Development Support Laboratory (LADETEC), Federal University of Rio de Janeiro (UFRJ), Cidade Universitária, Rio de Janeiro, 21941-598 Brazil; 2 Laboratory of Advanced Analysis in Biochemistry and Molecular Biology (LAABBM), Department of Biochemistry, Federal University of Rio de Janeiro (UFRJ), Cidade Universitária, Rio de Janeiro, 21941-909 Brazil; 3 Graduate Program in Biochemistry (PPGBq), Institute of Chemistry (IQ), Federal University of Rio de Janeiro (UFRJ), Cidade Universitária, Rio de Janeiro, 21941-909 RJ Brazil; 4 Analytical and Molecular Laboratory Center (CLAn), Institute of Chemistry (IQ), Federal University of Rio de Janeiro (UFRJ), Cidade Universitária, Rio de Janeiro, 21941-909 Brazil; 5 Graduate Program in Veterinary Hygiene (PGHIGVET), Faculty of Veterinary Medicine, Fluminense Federal University (UFF), Vital Brazil Filho, Niterói, 24230-340 RJ Brazil; 6 Graduate Program in Food Science, Institute of Chemistry, Federal University of Rio de Janeiro, Rio de Janeiro, RJ Brazil

## Abstract

Plasmids are major vectors driving the environmental dissemination of antimicrobial resistance (AMR) and other stress-resistance traits. The convergence between AMR and tolerance to multiple environmental stressors has become increasingly concerning, as these interactions intensify horizontal gene transfer and enhance plasmid conjugation. In this study, we investigated whether the co-occurrence of resistance determinants against different stressors results from random aggregation or statistically meaningful associations. We analyzed 25,116 complete plasmids from PLSDB and applied multivariate correspondence analysis to examine relationships between incompatibility groups and resistance categories. Pairwise co-occurrence patterns among resistance genes were assessed using Fisher’s exact test to determine whether their distribution deviated from randomness. IncF and IncH plasmids emerged as the incompatibility groups most strongly enriched in multidrug resistance, showing a marked tendency to co-carry genes conferring tolerance to antimicrobials, biocides, and metals-traits highly relevant under environmental co-selection. While pairwise tests did not reveal significant associations between specific gene pairs, the broader patterns of resistance accumulation highlight the structural evolution of plasmids via multireplicon cointegration as a primary mechanism for multi-stressor resistance. Our findings underscore the ecological importance of multireplicon plasmids, particularly those involving IncF and IncH, as high-risk vectors that sustain multi-stressor resistance in microbial communities.

**Supplementary Information:**

The online version contains supplementary material available at 10.1007/s00284-026-04819-z.

## Introduction

The increasing number of infections caused by multidrug-resistant bacteria has created a “silent pandemic,” progressively threatening the effectiveness of conventional antimicrobials (World Health Organization, [1]). In 2021, antimicrobial resistance (AMR) directly caused more than 1.14 million deaths and was associated with an additional 4.71 million deaths globally [2]. If current trends persist and effective control measures are not strengthened, projections indicate that antimicrobial resistance could result in approximately 39 million cumulative deaths worldwide between 2025 and 2050, with annual deaths directly attributable to AMR increasing substantially by mid-century [2].

Plasmids play a central role in this scenario as mobile genetic elements (MGEs) that mediate the horizontal transfer of antimicrobial resistance genes (ARGs) across diverse ecological niches [3]. Their maintenance and persistence in bacterial communities are strongly influenced by environmental pressures, including contamination by toxic and potentially toxic metals such as lead (Pb), mercury (Hg), arsenic (As), and chromium (Cr) [4]. These compounds exert direct selective pressure and act as synergistic drivers of AMR by enhancing horizontal gene transfer and promoting the spread of high-risk plasmids [5, 6].

Bacterial adaptation to combined selective pressures, including antimicrobials, metals, and biocides, relies primarily on three co-selection mechanisms: co-resistance, cross-resistance, and co-regulation [7]. Co-resistance occurs when resistance genes to multiple stressors are simultaneously present in the same MGEs, so transferring this vector ensures joint inheritance of the different resistances [8]. Cross-resistance occurs when a single mechanism, often multisubstrate efflux pumps, confers tolerance to several agents simultaneously [9, 10]. Co-regulation, in turn, is characterized by coordinated transcription under the control of the same regulatory protein or a shared promoter, which amplifies resistance to different agents [11]. Co-resistance plasmids, particularly those carrying both metal resistance genes (MRGs) and ARGs, exhibit high conjugation frequencies and represent a major challenge for management of AMR dissemination [12]. Several MRG-ARG co-occurrences have been reported in environmental and clinical isolates, including *czcA* with *sul1*, *sul2*, and *tet*(G); *merA* with *sul1*; *copA* or *pcoA* with *bla*_TEM_; and *pcoA* with *tet(G)* [8].

Although these observations suggest strong co-selection, much of the evidence remains correlational. Network-based analyses have identified recurrent MRG–ARG combinations, but their biological significance remains uncertain due to inherent limitations in sampling design, environmental heterogeneity, and biases toward specific classes of antimicrobials or metals [8]. Critically, few studies have employed statistical tests capable of distinguishing random co-occurrence from meaningful genetic association or have evaluated how plasmid incompatibility groups structure the distribution of multi-stressor resistance [8, 13, 14].

This study takes a novel approach by using multiple correspondence analysis (MCA) and Fisher’s exact test to distinguish meaningful associations between resistance genes and stressors such as metals and biocides, moving beyond random correlations. Through this methodology, we identify not only the co-occurrence of resistance determinants but also the types of plasmids mediating their simultaneous transport, emphasizing the importance of multireplicon plasmids. These findings represent a strategic opportunity to uncover new therapeutic targets and explore alternative strategies to combat AMR.

## Methodology

### Acquisition and Filtering of Plasmid Genomic Sequences

On June 25, 2025, 72,556 plasmid genomic sequences were retrieved from the PLSDB (Plasmid Database), https://ccb-microbe.cs.uni-saarland.de/plsdb/. Of these, 29,758 sequences were initially pre-selected based on annotations provided by the AMRFinderPlus tool, available on the web server. Subsequently, plasmid sequences annotated exclusively with virulence-associated genes were discarded, as this study focuses on resistance-driven plasmid ecology. Plasmids carrying resistance determinants were retained irrespective of the presence of virulence factors, ensuring that downstream analyses captured resistance accumulation and multi-stressor tolerance rather than pathogenicity-related traits. This filtering step resulted in a final dataset of 25,116 plasmid sequences included in this study. The annotations related to resistance genes (AMRFinderPlus) and plasmid incompatibility profiles (PlasmidFinder), used in this study, are obtained directly from the PLSDB repository.

### Statistical analysis

#### Statistical Analysis of the Proportion of Multidrug-Resistant Plasmids by Incompatibility Group

The proportions of multidrug-resistant (MDR, defined as carrying ≥ 3 distinct resistance classes) plasmids in the ten most prevalent incompatibility groups (Inc) were evaluated. The proportion of MDR plasmids in each group was calculated as:

$${\rm{MDR\, proportion}} = \frac{Number\, of\,MDR\,plasmids}{Total\,plasmids\,of\,the\,Inc\,group}$$

For each Inc group, mean, standard deviation (SD), and standard error of the mean (SE) were estimated. Because the MDR variable is binary, normality was not assumed. Differences between groups were assessed using the Kruskal-Wallis test and Dunn’s post hoc test with the Bonferroni correction for multiple comparisons. The post-test revealed statistically distinct pairs of groups (*p* < 0.05), which were represented in the graph by significance letters. The analyses were performed in R (version 4.0.0) with the ggplot2, dplyr, and rstatix packages [17].

Multireplicon plasmids were decomposed into their individual incompatibility groups for analysis. Each replicon type was treated independently and associated with the resistance profile of the plasmid in which it occurred, allowing replicon-level assessment of resistance enrichment irrespective of co-occurring replicons.

#### Statistical Analysis of the Relationship between the Number of Replicons and Resistance Genes

The relationship between the number of plasmid replicons and resistance gene load was evaluated based on 21,911 resistant sequences deposited in the PLSDB. Replicons were identified using PlasmidFinder data, and resistance genes were extracted from *gene_symbol* annotations in the AMRFinderPlus spreadsheet. The total number of resistance genes was determined for each sequence, and plasmids were grouped according to the number of replicons detected (*n_typing*).

The average number of resistance genes per sequence in each group was calculated as:

$$\underset{\_}{{x}_{i}}=\frac{1}{{n}_{i}}{\sum}_{j=1}^{{n}_{i}}{x}_{ij}$$

Where *x*_*ij*_​ corresponds to the number of genes in sequence *j* with *i* replicons, and *n*_*i*_​ is this group’s total number of plasmids. The standard error of the mean (SE) was estimated as:

$${\rm SE} = \frac{SD}{\sqrt{{n}_{i}}}$$

To test for differences between groups, the Kruskal-Wallis test was applied, followed by Dunn’s post-hoc test with the Bonferroni correction for multiple comparisons. All analyses were performed in R (version 4.4.0) with the ggplot2, dplyr, and rstatix packages.

#### Correspondence analysis (CA)

Correspondence Analysis (CA) was used to quantify the association between incompatibility groups (Inc) and resistance categories, taking the plasmid as the sampling unit. For plasmids with multiple replicons, each replicon was considered present, and resistance categories were defined by the presence of at least one gene of the class. An Inc × Category contingency matrix with plasmid counts was constructed; rows and columns with zero frequency were excluded, and rare residual categories were omitted from the main biplot. Classical CA (chi-square distance over row and column profiles) was applied.

#### Fisher’s exact test

For each plasmid, we constructed binary presence/absence matrices: (i) genes (restricted to the most prevalent for stability) and (ii) resistance categories (antibiotic, biocide, metal). For each pair of variables (gene-gene or category-category), we formed a 2 × 2 (*a*, *b*, *c*, *d*) co-occurrence table by plasmid. We applied the two-tailed Fisher’s exact test, estimating the odds ratio OR = $\frac{ad}{bc}$ and its 95% CI (extracted from Fisher’s own test). For interpretation and visualization, we used log_2_ (OR) (positive = co-occurrence; negative = anticorrelation). *p*-values ​​were adjusted by Benjamini-Hochberg (FDR), retaining associations with q < 0.05 and log_2_ (OR) ≥ 0.5 (around OR ≥ 1.41 or ≤ 0.71). Pairs outside these criteria were masked (NA). The HCA was constructed from a symmetric filtered log2(OR) matrix, using agglomerative hierarchical clustering (Euclidean distance and average linkage/UPGMA), to ensure that the heatmap displays only associations not attributable to chance and with a relevant magnitude. The corresponding Fisher’s exact test results are reported in Supplementary Table S1 (Fisher test).

#### Data Availability

All R scripts and processed datasets required to reproduce the correspondence analysis and pairwise resistance gene association heatmap presented in this study are publicly available at: https://github.com/hannaycasouza-maker/plasmid_replicon_association_analysis.

## Results

### Overview of plasmid genomic sequences

Of the 72,556 plasmid genome sequences deposited in the PLSDB repository, 25,116 corresponded to resistant plasmids, representing approximately 34.62%. Initially, we sought to characterize the resistance profile within the plasmids; for this, we defined categories based on the stress factors associated with each type of resistance.

#### General characterization of resistant plasmids

**Fig. 1:**
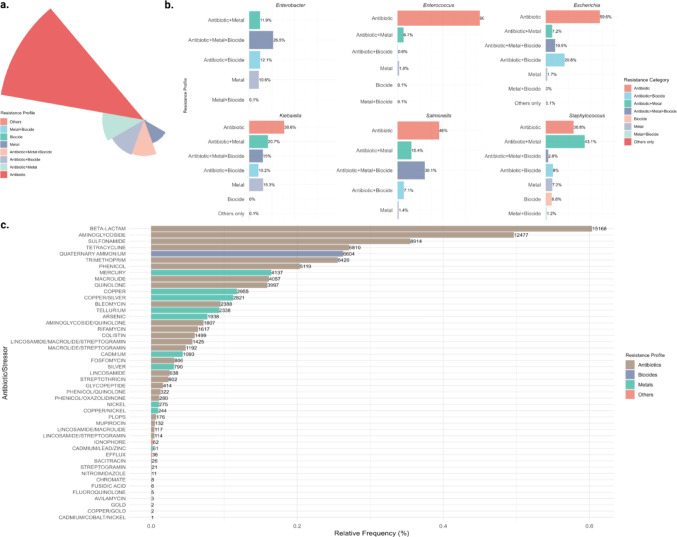
Antimicrobial resistance profiles and relative distribution of resistance classes. (**a**) Circular barplot showing the overall distribution of resistance profiles by category; **(b)** Clustered bar graphs showing the resistance profile among the most common bacterial genera, including *Enterobacter*, *Klebsiella*, *Enterococcus*, *Escherichia*, *Salmonella*, and *Staphylococcus*. Each graph displays the relative frequency of resistance categories (antibiotic, antibiotic + metal, antibiotic + biocide, antibiotic + metal + biocide, metal, biocide, metal + biocide, and others) within the respective genera; **(c)** A horizontal bar graph illustrates the relative distribution of resistance classes associated with antibiotics and other stressors. The analysis is based on a sample of 25,116 unique accessions, and values are presented as relative frequency percentages. Resistance categories are colored according to the indicated legend, with antibiotics in brown, biocides in purple, metals in green, and others in salmon. PLOPS, Phenicol/Lincosamide/Oxazolidinone/Pleuromutilin/Streptogramin

The established categories are: (i) Antibiotic; (ii) Antibiotic + Biocide; (iii) Antibiotic + Metal; (iv) Antibiotic + Metal + Biocide; (v) Biocide; (vi) Metal; (vii) Metal + Biocide; and (viii) Others, including resistance related to structural mechanisms, such as the presence of efflux pumps. Most plasmid sequences (*n =* 12,680, 50.49%) exhibited resistance only to antimicrobials. Combined resistance to antimicrobials and metals was observed in (*n =* 3,649, 14.53% of plasmids). Other resistance patterns were less frequent, such as resistance to antimicrobials and biocides (*n =* 3,232, 12.87%), followed by resistance to antimicrobials, metals, and biocides simultaneously (*n =* 3,145, 12.52%). Isolated resistances to biocides (*n* = 196, 0.78%) and metals (*n =* 2,169, 8.64%) were also detected, while combinations of metals and biocides represented a small fraction (*n =* 31, 0.12%). A minimal number of plasmids (*n =* 14, 0.06%) exhibited resistance only to factors related to structural mechanisms, such as efflux pumps (Fig. 1a).

#### Distribution of Antimicrobial Resistance Classes in Plasmid Sequences

The resistance profile was analyzed according to previously defined categories. It was observed among the ten most common bacterial genera, with the following plasmids isolated: *Klebsiella* (*n =* 7,664), *Escherichia* (*n =* 6,487), *Staphylococcus* (*n =* 2,044), *Salmonella* (*n =* 1,624), *Enterococcus* (*n =* 1,324), *Enterobacter* (*n =* 1,232), *Acinetobacter* (*n =* 819), *Citrobacter* (*n =* 638), *Pseudomonas* (*n =* 384), and *Shigella* (*n =* 347), as detailed in Supplementary Table S2 (Resistance Category by Gender). However, only data from the six most frequent genera were presented for graphical visualization, as shown in Fig. 1b.

Subsequently, the absolute and relative frequencies of plasmid sequences were analyzed to better characterize the resistance profile, according to their distribution among the resistance classes that comprise the previously defined categories (Fig. 1c). Furthermore, the distribution of resistance classes among the ten most prevalent bacterial genera is presented in the Supplementary Table S3 (Resistance Classes by Gender).

Despite their clinical relevance, carbapenems were grouped within the beta-lactam class to maintain analytical consistency across resistance categories. A dedicated analysis of carbapenemase-encoding plasmids has been previously published [18].

### Overview of Incompatibility Profiles in Bacterial Plasmids

Based on the data available through the PlasmidFinder tool, the incompatibility profile of the plasmid sequences present in the analyzed set was characterized. In total, 40,208 plasmid sequences had PlasmidFinder data available. Of these, 21,911 (54.5%) contained at least one resistance gene, while 18,297 (45.5%) had no identifiable resistance genes.

**Fig. 2:**
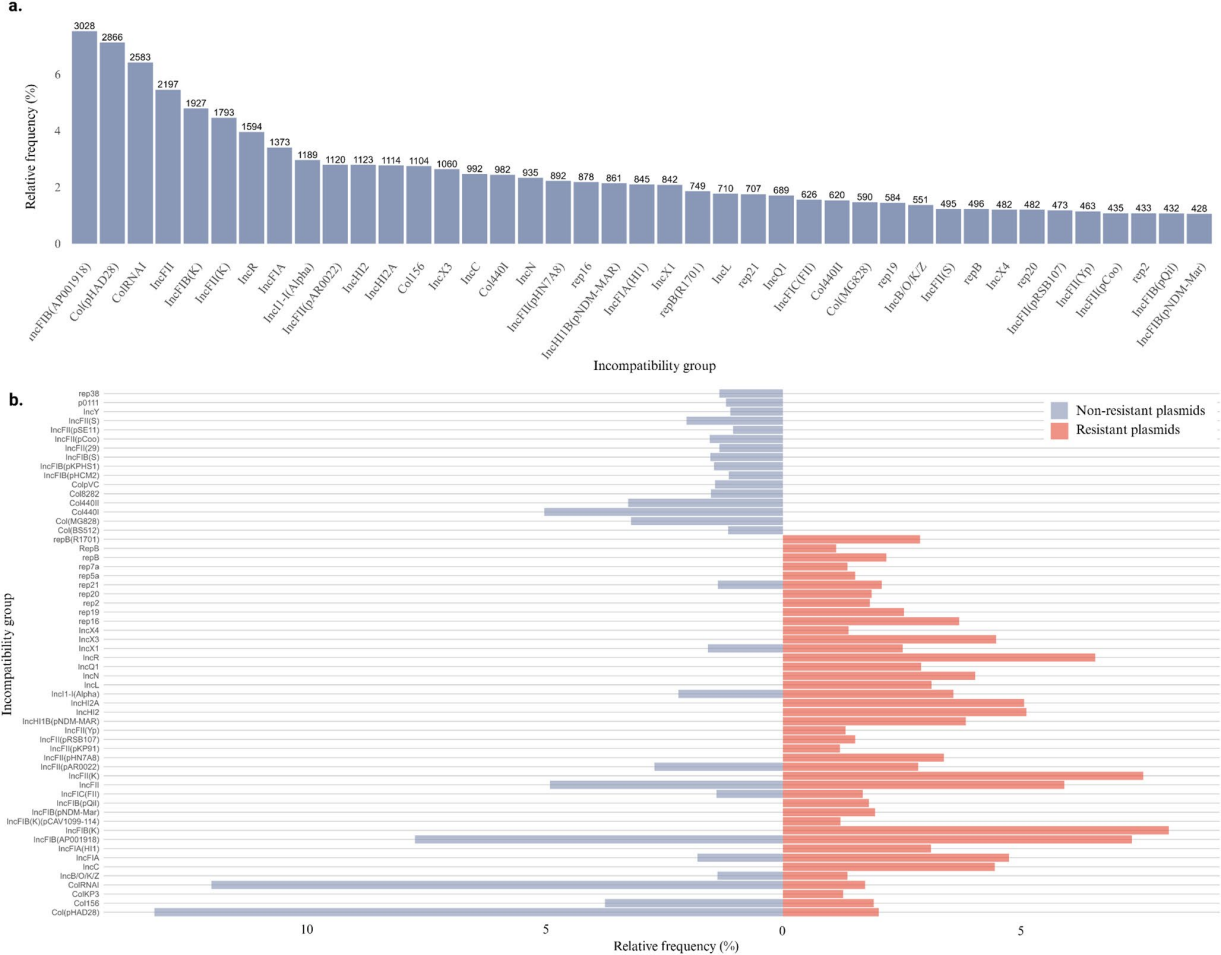
Distribution of the most prevalent plasmid incompatibility groups in the analyzed data. **(a)** Overall relative frequency (%) of the most representative incompatibility groups, calculated using the complete set of plasmid sequences as the denominator. **(b)** Relative frequency (%) of incompatibility groups stratified by resistance status, with frequencies calculated independently within resistant (red) and non-resistant (blue) plasmid subsets

The initial analysis was based on the distribution of incompatibility groups, considering the total set of sequences, without taking into account the occurrence of multiple replicons in the same plasmid sequence (Fig. 2a). Absolute frequencies were calculated based on the individual presence of each incompatibility group per sequence, so the relative frequency represents the proportion of sequences that present a given incompatibility group in relation to the total sequences analyzed.

#### Comparison of the incompatibility profile between resistant vs. non-resistant plasmids

Then, the incompatibility groups were compared between the resistant and non-resistant sequences to observe possible association trends between certain incompatibility groups and the presence of resistance genes (Fig. 2b).

### Association between Resistance and Incompatibility

In our data, the most frequent incompatibility groups among resistant sequences were: IncFIB(K) (*n =* 1,781), IncFII(K) (*n =* 1,664), IncFIB(AP001918) (*n =* 1,610), IncR (*n =* 1,442), IncFII (*n =* 1,299), IncHI2 (*n =* 1,123), IncHI2A (*n =* 1,114), IncFIA (*n =* 1,044), IncX3 (*n =* 984), and IncC (*n =* 977).

#### Incompatibility profile between resistant sequences

Based on the taxonomic classification of resistant bacterial isolates, the distribution of plasmid incompatibility groups varied across genera and species. At the genus level, several incompatibility groups were shared among multiple bacterial genera, indicating the presence of plasmid backbones with broad host ranges. In contrast, some incompatibility groups showed a more restricted distribution, being predominantly associated with specific taxa. Notably, high relative frequencies of specific incompatibility groups were observed in *Acinetobacter* (0.999) and *Pseudomonas* (0.898), whereas *Enterococcus* (rep2, 0.300) and *Staphylococcus* (rep16, 0.394) exhibited more moderate associations. At the species level, a similar pattern was observed, with both widely distributed and taxon-associated incompatibility groups identified. The complete distributions are provided in Supplementary Tables S4 and S5.

#### Distribution of Resistance Categories as a Function of Incompatibility Profiles

**Fig. 3:**
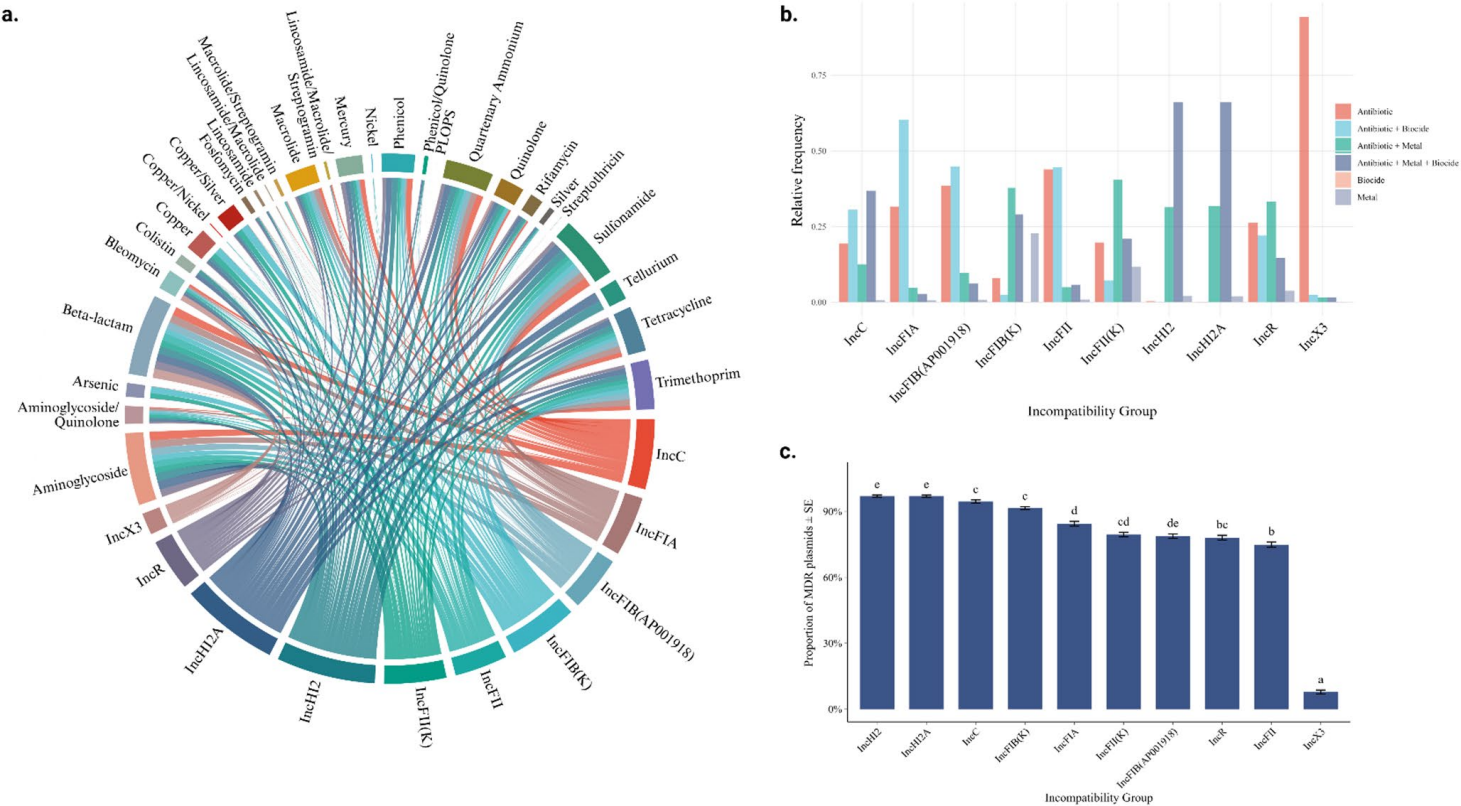
Distribution of resistance among the most prevalent plasmid incompatibility groups. **(a)** Chord diagram representing the association between the ten most prevalent plasmid incompatibility groups (Inc) and the different resistance classes in the resistant plasmids. The connections (chords) illustrate the co-occurrence of each Inc group with the respective resistance classes, considering unique plasmids. The thickness of the chords reflects the relative frequency of the association between each incompatibility group and the resistance class. **(b)** Relative frequency of resistance categories (Antibiotic; Antibiotic + Biocide; Antibiotic + Metal; Antibiotic + Metal + Biocide; Biocide and Metal) among the ten most frequent incompatibility groups. **(c)** Mean proportion of multidrug-resistant (MDR) plasmids associated with the ten most prevalent incompatibility groups (Inc), with respective standard errors of the mean (± SE). Plasmids harboring genes associated with resistance to three or more classes of stressors were considered MDR. The analyses were performed based on unique plasmids (NUCCORE_ACC) in the PLSDB database. Significant differences between groups were determined by the Kruskal-Wallis test (χ² = 4010.415; df = 9; *p* < 0.0001), followed by Dunn’s test with Bonferroni correction (*p* < 0.05). Different letters indicate statistically significant differences between groups. PLOPS, Phenicol/Lincosamide/Oxazolidinone/Pleuromutilin/Streptogramin

The distribution of predefined resistance categories (Antibiotic; Antibiotic + Biocide; Antibiotic + Metal; Antibiotic + Metal + Biocide; Biocide and Metal) was analyzed among the most frequent incompatibility groups in the resistant sequences, to identify possible trends related to the co-occurrence of resistance determinants in specific incompatibility groups (Fig. 3b).

#### Associations between incompatibility profiles and resistance classes

To assess the existence or absence of co-occurrence patterns between plasmid types and resistance classes, we evaluated possible associations between different resistance classes and the ten most frequent incompatibility groups among resistant sequences (Fig. 3a). Subsequently, an analysis was performed to observe the multidrug resistance profile associated with the ten most prevalent plasmid incompatibility groups, aiming to identify the frequency with which these incompatibility groups are involved in the simultaneous carriage of multiple resistance classes (Fig. 3c). The global test indicated significant differences (*x*^2^ = 4010.415, df = 9, *p* < 2.2 × 10⁻¹⁶). The post-test revealed statistically distinct pairs of groups (*p* < 0.05), which are represented in the graph by significance letters. The means ± SE were represented in a bar graph (Fig. 3c), in which groups with a lower proportion of MDR (e.g., IncX3) and others with significantly higher values (e.g., IncHI2A and IncHI2) stood out.

### Association between Number of Replicons and Resistance Categories

Based on the 21,911 resistant plasmid sequences with available data in PlasmidFinder, the distribution of resistance categories was analyzed according to the number of replicons present in each plasmid (Table 1). The objective was to investigate whether plasmids with multiple replicons are more likely to carry simultaneous resistance to various stressors.

**Table 1:** Distribution of Resistance Categories as a Function of the Number of Replicons

Number of replicons	Category	Count	Relative Frequency (%)
1	*Antibiotic*	7869	69%
	*Antibiotic + Biocide*	1414	12%
	*Antibiotic + Metal*	554	5%
	*Antibiotic + Metal + Biocide*	894	8%
	*Biocide*	86	1%
	*Metal*	612	5%
	*Metal + Biocide*	1	0%
2	*Antibiotic*	1771	24%
	*Antibiotic + Biocide*	656	9%
	*Antibiotic + Metal*	2357	32%
	*Antibiotic + Metal + Biocide*	1396	19%
	*Biocide*	55	1%
	*Metal*	1055	14%
	*Metal + Biocide*	22	0%
3	*Antibiotic*	656	27%
	*Antibiotic + Biocide*	515	21%
	*Antibiotic + Metal*	546	22%
	*Antibiotic + Metal + Biocide*	525	21%
	*Metal*	210	9%
	*Metal + Biocide*	5	0%
4	*Antibiotic*	163	26%
	*Antibiotic + Biocide*	236	37%
	*Antibiotic + Metal*	87	14%
	*Antibiotic + Metal + Biocide*	129	20%
	*Biocide*	1	0%
	*Metal*	19	3%
5	*Antibiotic*	11	17%
	*Antibiotic + Biocide*	15	24%
	*Antibiotic + Metal*	12	19%
	*Antibiotic + Metal + Biocide*	20	32%
	*Metal*	5	8%
6	*Antibiotic*	2	14%
	*Antibiotic + Biocide*	5	36%
	*Antibiotic + Metal*	3	21%
	*Antibiotic + Metal + Biocide*	4	29%

#### Resistance and Incompatibility Profiles According to the Number of Replicons

**Fig. 4:**
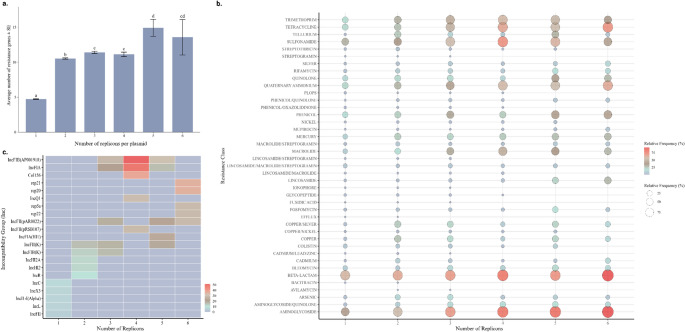
Relationship between the number of replicons per plasmid, gene carriage, and resistance profiles. **(a)** Mean number of resistance genes per plasmid (± standard error) as a function of the number of replicons. Different letters indicate statistically significant differences between groups (Dunn’s test, *p* < 0.05). **(b)** Distribution of resistance classes in plasmids containing 1 to 6 replicons. The size and color of the circles represent, respectively, the relative frequency and intensity per class. **(c)** Relative frequency of the main plasmid incompatibility groups (Inc) associated with different numbers of replicons. PLOPS, Phenicol/Lincosamide/Oxazolidinone/Pleuromutilin/Streptogramin

The proportion of resistance genes was determined based on the number of replicons in each plasmid (Fig. 4a). The average number of resistance genes per plasmid varied significantly according to the number of replicons (Kruskal-Wallis, χ² = 4684.2; df = 5; *p* < 2.2 × 10⁻¹⁶). Plasmids with only one replicon exhibited the lowest gene load, differing from all other groups (*p* < 0.001) (Fig. 4a). Those containing two replicons presented intermediate values, significantly higher than the group with one replicon, but lower than the groups with three and five replicons (*p* < 0.05) (Fig. 4a). No significant differences were observed among plasmids with three to six replicons, indicating a plateau in the resistance load in plasmids with multiple replicons. Means and standard errors were plotted with significance letters indicating statistical groupings (Fig. 4a).

In addition, the distribution of resistance classes was also observed, allowing us to assess the diversity of resistance classes according to plasmid structure (Fig. 4b). Finally, we investigated which incompatibility groups were most frequently associated with resistant plasmids containing multiple replicons (Fig. 4c).

### Correspondence analysis (CA)

**Fig. 5:**
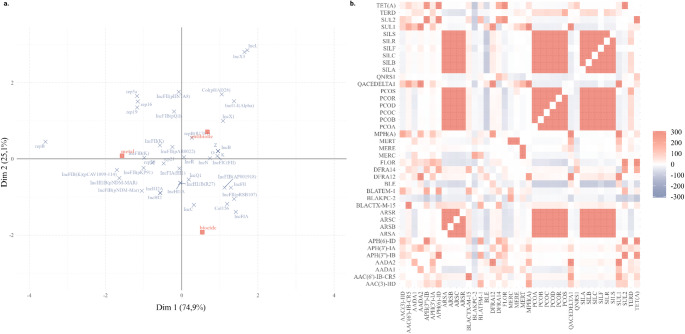
Multivariate analysis of the association between plasmid incompatibility groups and antimicrobial resistance determinants and environmental stressors. **a**. Multiple correspondence analysis (MCA) representing the distribution of different plasmid replicons (blue crosses) in relation to the presence of antibiotic, metal, and biocide resistance genes. The principal axes (Dim 1 and Dim 2) explain 74.9% and 25.1% of the total variance, respectively; **b.** Correlation matrix between resistance genes, presented as a heat map, indicating positive (red) and negative (blue) associations between the different determinants, with intensity proportional to the normalized values

Correspondence analysis (CA) of plasmid incompatibility groups against resistance categories resolved 100% of the total inertia in two dimensions (Dim1 = 74.9%, Dim2 = 25.1%) (Figure 5a). The first axis captured the dominant gradient, opposing metal-associated replicons on the positive side from antibiotic-associated groups on the negative side, while the second axis separated biocide determinants toward negative values. The category ‘others’ remained close to the centroid, indicating a limited directional association.

Among the replicons, IncHI1B(pNDM-Mar), IncHI2A, and IncFIB(K) were strongly aligned with the metal pole, reflecting their preferential occurrence in plasmids carrying metal resistance determinants (Figure 5a). Conversely, IncX3 and IncL localized toward the antibiotic pole, consistent with their frequent presence in plasmids enriched for antibiotic resistance genes (Figure 5a). In the lower left quadrant, IncFIA, IncFII, and IncFIB(AP001918) projected closer to the biocide vector, suggesting an affinity with plasmids harboring tolerance determinants to biocides (Figure 5a). Replicons, such as IncN, IncQ1, and IncC, clustered around the origin, exhibiting weak or heterogeneous associations.

Together, the ordination delineates a clear structural organization: a metal-anchored module dominated by IncHI and IncFIB(K) contrasted with an antibiotic/biocide module characterized by IncX, IncL, and IncFI-family replicons, underscoring distinct selective pressures shaping plasmid backbones across resistance categories.

## Discussion

In this study, we conducted a large-scale, plasmid-centered analysis to examine the co-occurrence of resistance determinants to antibiotics, metals, and biocides across diverse bacterial taxa. Our findings demonstrate that plasmid-mediated resistance patterns are not primarily governed by deterministic gene–gene associations, but instead emerge from higher-order plasmid architectures, particularly multireplicon backbones and incompatibility group structure. Notably, non-antibiotic selective pressures, such as exposure to metals and biocides, appear to play a central role in shaping plasmid persistence and resistance enrichment, emphasizing plasmids as integrated evolutionary units that mediate co-selection across multiple stressors.

Plasmids carrying exclusively ARGs constituted the most frequent pattern, reflecting the intense selective pressure exerted by the inappropriate use of antibiotics. This observation is consistent with previous reports indicating the predominance of ARG-only plasmids among resistant backbones [19]. Global increases in antimicrobial consumption in both regulated and unregulated production systems have intensified selective pressures favoring plasmid-mediated resistance [20]. Furthermore, sustained antimicrobial use in both clinical and agricultural settings reinforces selection for plasmid-mediated resistance, accelerating the emergence and dissemination of multidrug-resistant bacterial populations [21, 22].

Persistent metal contamination arising from mining, industrial, and agricultural activities creates selective pressures that promote co-selection between ARGs and MRGs [23, 24]. Although estimates from the European Environment Agency [25] indicate a reduction in emissions of cadmium (Cd), mercury (Hg), and lead (Pb) since 1990, these contaminants remain a significant environmental challenge (European Environment Agency, [25]). Recent data indicate that between 14 and 17% of global agricultural lands have toxic metal pollution levels above safe thresholds [26]. Under these conditions, plasmids carrying metal resistance operons, such as arsenic (*arsB*, *arsC*, and *arsR*), copper (*pcoABCDER* and *copABCLRS*), and mercury (*merA*, *merB*, and *merT*), are preferentially maintained, as observed in the analyzed plasmid sequences, facilitating the co-selection and persistence of antimicrobial resistance through co-resistance mechanisms [27, 28].

Resistance to quaternary ammonium compounds (QACs) was more prevalent than resistance to other environmental stressors, including metals. The unexpectedly high prevalence of QAC resistance observed in our dataset suggests that biocide-driven selection may be systematically underestimated in plasmid-focused AMR surveillance. The widespread application of QACs in hospital, industrial, and domestic environments likely contribute to this increase, reflecting a selective pressure often overlooked in studies addressing plasmid-mediated resistance. These compounds, widely used as antiseptics, disinfectants, preservatives, and antimicrobial agents in healthcare settings, the food-processing industry, personal hygiene products, and household cleaning formulations, create continuous low-level exposure that favors the persistence and selection of tolerant or resistant bacterial populations (Merchel Piovesan Pereira and Tagkopoulos, 2019). The prolonged presence of subinhibitory concentrations of these compounds in the environment can favor the selection of bacteria that are tolerant and resistant [29, 30]. The emergence of this resistance has been documented in both clinical isolates, such as *Staphylococcus aureus*, *Escherichia coli*, *Klebsiella pneumoniae*, and *Pseudomonas aeruginosa*, and foodborne pathogens, including *Listeria monocytogenes*, posing an additional challenge for infection control and food safety [31].

Previous studies have reported frequent co-occurrence and genetic linkage between metal resistance genes and ARGs, including associations involving mercury, arsenic, copper, and clinically relevant antibiotics [8, 11]. Genetic linkage between arsenic or mercury resistance and antibiotic resistance has also been demonstrated in *Serratia marcescens* [11], as well as in the conjugative plasmid pHCM1, which co-harbors *mer* operons and determinants for chloramphenicol, ampicillin, streptomycin, sulfonamide, and trimethoprim resistance [11, 32]. Similarly, megaplasmids from *Salmonella enterica*, such as pESI and the structurally related pESM described in our previous studies, exemplify large multireplicon backbones that concomitantly carry mercury resistance operons (*mer*), biocide tolerance genes including *qacEΔ1* and other QAC-associated determinants, and an extensive repertoire of antimicrobial resistance genes. These megaplasmids demonstrate how co-selection pressures exerted by metals, sanitizing agents, and antimicrobials can stabilize exceptionally complex resistance platforms and facilitate their dissemination across diverse ecological niches [33–35].

In contrast, our large-scale analysis did not reveal consistent positive correlations between individual ARG-MRG gene pairs (Figure 5b). Rather than contradicting the relevance of co-resistance, this finding underscores a central insight of this study: resistance co-occurrence is structured at the plasmid level, not by deterministic gene–gene associations. ARGs and MRGs can be stably maintained and disseminated when physically linked on the same plasmid, independent of detectable pairwise gene-level correlations [9]. Thus, the absence of strong gene-to-gene correlations does not diminish the role of plasmids as key vehicles for the joint spread of multiple resistance determinants.

Although the large number of resistance genes and the diversity of bacterial populations complicate the detection of clear correlations, our study has highlighted the structural mechanisms of plasmids as key drivers in the co-occurrence of resistance determinants. However, as noted, the complexity of these genetic interactions requires careful consideration of potential methodological limitations. It is important to emphasize that while our study adopted a conservative approach by applying the Bonferroni correction to control for Type I errors, the high genetic diversity within *Enterobacteriaceae* populations, coupled with the large number of resistance genes considered, may have diluted statistical signals, hindering the detection of significant associations. We acknowledge this limitation in our analysis and underscore the need for alternative strategies, such as reducing the number of comparisons or employing less conservative methods, to address the genetic complexity of these populations. Such approaches could potentially uncover clearer associations between resistance genes, offering more insight into the underlying mechanisms of antimicrobial resistance.

The co-occurrence of antimicrobial resistance genes with genes conferring tolerance to other environmental stressors, such as heavy metals and biocides, within the same plasmid backbone can be explained by well-documented plasmid structural and evolutionary mechanisms. These include physical linkage of resistance determinants within the same replicon, integration mediated by mobile genetic elements such as transposons, integrons, and insertion sequences, and cointegration events generating multireplicon plasmid backbones [36–38]. In addition, sustained exposure to overlapping selective pressures, particularly antibiotics, metals, and biocides, promotes co-selection and long-term maintenance of these composite resistance platforms [9, 39, 40]. In this context, plasmids function as adaptive genetic platforms that facilitate the joint persistence and horizontal dissemination of multiple resistance determinants. The physical linkage of antimicrobial resistance genes with genes conferring tolerance to metals and biocides is primarily driven by transposons, which act as mobile genetic elements capable of relocating within and between DNA molecules [37]. These elements function as discrete units carrying multiple genes and their associated promoters, and their movement is mediated by transposases that catalyze excision and integration events [37]. When selection favors a single gene within a transposon, the entire genetic unit is maintained, resulting in co-selection of adjacent resistance genes even in the absence of direct selective pressure for each determinant individually [36, 37].

In our dataset, plasmids from the IncF and IncH incompatibility groups were the most frequent. IncF plasmids have been widely reported in the literature as the most frequent, and their occurrence is particularly high among resistant plasmids [41, 42]. Similarly, IncH plasmids, especially the IncHI2 subtype, are frequently associated with the carriage of antibiotic and heavy metal resistance genes, being predominant in *Salmonella* isolates and other multidrug-resistant strains, surpassing other groups such as IncA/C and IncI [41, 43].

The ecological distribution of these groups, however, is not uniform across environments or hosts. For example, among *E. coli* isolated from surface waters in Tanzania, IncF was detected in 49% of samples, while IncHI2 was present in only 6% of isolates, demonstrating ecological differences in the distribution of these plasmids [44]. Although IncH plasmids are often described as being primarily associated with *Enterobacteriaceae*, available evidence indicates that their potential host range extends beyond this family, encompassing a broader spectrum of Gram-negative bacteria, including aquatic and food-associated species such as *Aeromonas salmonicida*, *Vibrio anguillarum*, and *Yersinia ruckeri* (Rozwandowicz et al., 2018). This contrasts with IncF plasmids, whose host range is largely restricted to *Enterobacteriaceae* despite their high prevalence in clinical settings (Rozwandowicz et al., 2018).

Overall, the predominance of IncF and IncH plasmids reflects distinct but complementary strategies. IncF plasmids combine efficient conjugation and functional flexibility within *Enterobacteriaceae* through multireplicon architectures (e.g., FII, FIA, and FIB), which mitigate incompatibility constraints and favor resistance accumulation [42, 45]. In contrast, the persistence of IncH plasmids is facilitated by their frequent occurrence as multireplicon backbones and their enrichment in mobile genetic elements, which promote cointegration and the accumulation of resistance determinants (Caratolli, 2009; [46]).

IncH plasmids are typically large and structurally complex, with a high capacity to accumulate multiple replicons and resistance determinants [47]. This architectural flexibility likely contributes to the frequent co-occurrence of antimicrobial resistance, metal tolerance, and biocide resistance genes observed in this incompatibility group [37, 38]. Additionally, the temperature-dependent conjugation of IncH plasmids, with optimal transfer occurring at lower temperatures, allows these plasmids to persist in environmental, food-related, and animal-associated reservoirs, thereby expanding their ecological reach (Rozwandowicz et al., 2018). Together, these features help explain the epidemiological success of IncH plasmids alongside IncF plasmids. Ultimately, the ability of multireplicon architectures to overcome incompatibility constraints, coupled with the resilience of IncH plasmids despite their higher metabolic cost, underscores the success of both plasmid types in different ecological contexts.

Although direct analysis did not reveal predominant associations between incompatibility groups and specific resistance classes, correspondence analysis (CA) indicated significant patterns of association between certain replicons and resistance categories (metals, antibiotics, and biocides). Plasmids belonging to the IncF incompatibility group, including subtypes IncFII(K), IncFIB(K), IncFIB(K)(pCAV1099-114), IncFII(pKP91), IncFIB(pNDM-Mar), as well as IncH plasmids, represented by subtypes IncHI1B(pNDM-MAR), IncHI2A, and IncHI2, clustered preferentially on the axis associated with metal resistance. In contrast, replicons of the IncFIB(AP001918), IncFII, IncFII(pRSB107), IncFIA, and IncHI1B(R27) subtypes exhibited greater proximity to the biocide-associated vector, suggesting that these plasmids have a selective propensity to carry genes related to resistance to disinfectants and non-antibiotic antimicrobial compounds.

Interestingly, despite the central role of IncF and IncH plasmids in the global dissemination of antibiotic resistance, these replicons did not align with the antibiotic-associated axis. This finding suggests that, although ARGs are commonly found on these plasmids, their maintenance and ecological success may be primarily driven by non-antibiotic selective pressures, particularly those related to metals and biocides [48]. This pattern may reflect the mechanism of adaptive co-selection, in which resistance to multiple distinct stressors increases the potential for persistence and dissemination of these plasmids in different ecological niches and selective contexts ([49]; Pal et al., 2015).

Our data indicate that the epidemiological success of IncF and IncH plasmids is driven not solely by antibiotic resistance, but by their capacity to act as platforms for multi-stressor co-selection. This functional versatility is closely linked to their frequent multireplicon architecture and the enrichment of mobile genetic elements, such as transposons and integrons, which facilitate the acquisition, integration, and stabilization of diverse resistance determinants [37]. As a result, multireplicon plasmids display broader multidrug resistance profiles and frequently encode resistance to multiple antimicrobial and environmental stressors. This pattern is evident in plasmids isolated from *Klebsiella* spp., where the coexistence of multiple replicon types is linked to increased accumulation of antimicrobial resistance genes, potentially enhancing host adaptability and persistence under selective pressure [19]. Collectively, these features position IncF- and IncH-dominated multireplicon plasmids as highly efficient vectors for the dissemination of multidrug resistance, underscoring their relevance as priority targets for epidemiological surveillance and intervention strategies.

Multireplicon plasmids represent highly dynamic genetic platforms that frequently arise through cointegration events involving distinct replicons, resulting in increasingly complex plasmid architectures. However, it remains unclear whether replicon multiplicity is a direct driver of resistance gene accumulation or a consequence of underlying evolutionary processes, such as frequent recombination, elevated mobile genetic element activity, and stress responses associated with plasmid transfer.

Previous studies suggest that plasmid cointegration and the increased prevalence of mobile genetic elements, including transposons (Tn) and integrons (In), may facilitate both the emergence of multireplicon structures and the accumulation of resistance genes through horizontal gene transfer [50]. In addition, conjugative plasmid transfer has been shown to induce the bacterial SOS response, which can activate integrons and promote resistance gene acquisition, potentially accelerating resistance gene accumulation on plasmids [51]. Together, these mechanisms suggest that multireplicon plasmids may represent the outcome of intense genetic exchange and stress-driven evolutionary processes, conferring enhanced adaptability under diverse environmental and clinical selective pressures [19].

Taken together, our findings demonstrate that the evolutionary success of IncF and IncH plasmids is not solely attributable to their association with ARGs, but rather to their ability to integrate and disseminate resistance to multiple classes of environmental stressors, especially metals and biocides. These stressors likely act as primary ecological drivers, sustaining plasmid persistence in diverse niches and functioning as powerful, yet often overlooked, forces in the long-term maintenance of the plasmid-mediated resistome. It should be noted that the present analysis was not stratified by host or source (human, veterinary, or environmental), as such metadata are frequently incomplete or inconsistently annotated in PLSDB. Accordingly, source-specific inferences were deliberately avoided, and interpretations were restricted to plasmid-level architectures and resistance patterns independent of host context. By revealing that two of the most epidemiologically relevant plasmid families are primarily shaped by non-antibiotic selection, our study highlights the need to expand AMR surveillance and mitigation strategies beyond the traditional focus on antibiotic stewardship. Targeting plasmid maintenance pathways, conjugative machinery, or cointegration processes represents a promising direction for precision interventions aimed at disrupting the hidden ecological engines that sustain multidrug resistance.

## Conclusion

Our findings demonstrate that co-resistance, rather than deterministic gene–gene associations, is the primary force sustaining multidrug resistance across bacterial populations. Multireplicon plasmids, particularly those from the IncF and IncH incompatibility groups emerge as highly efficient vectors, concentrating ARGs, MRGs and biocide-resistance genes and enabling their stable maintenance and horizontal dissemination. The unexpected association of IncF/IncH plasmids with metal and biocide resistance, rather than antibiotic determinants, underscores the role of non-antibiotic selective pressures as hidden drivers of plasmid persistence. These results underscore the need to expand AMR control strategies beyond antibiotic stewardship, incorporating approaches that target plasmid biology and environmental co-selectors.

## Supplementary Information

Below is the link to the electronic supplementary material.

Supplementary Material 1

## Data Availability

All data analyzed in this study were obtained from publicly available sources. A total of 72,556 plasmid sequences and their associated metadata were retrieved from the PLSDB database (https://ccb-microbe.cs.uni-saarland.de/plsdb/) in June 2025, when the database was last accessed for this study. No new sequencing data were generated. All analysis workflows and processed datasets are available from the corresponding author upon reasonable request.
